# The efficacy of eye movement desensitization and reprocessing in reducing anxiety among female university students with primary dysmenorrhea

**DOI:** 10.1186/s40359-022-00757-0

**Published:** 2022-03-03

**Authors:** Sahar Valedi, Mohammad MoradiBaglooei, Mehdi Ranjbaran, Venus Chegini, Mark D. Griffiths, Zainab Alimoradi

**Affiliations:** 1 Students Research Committee, School of Nursing and Midwifery, Qazvin University of Medical Sciences, Qazvin, Iran; 2 School of Nursing and Midwifery, Qazvin University of Medical Sciences, Qazvin, Iran; 3 School of Health, Qazvin University of Medical Sciences, Qazvin, Iran; 4 Obstetrics and Gynecology Department, School of Medicine, Qazvin University of Medical Sciences, Qazvin, Iran; 5 International Gaming Research Unit, Psychology Department, Nottingham Trent University, Nottingham, UK; 6 Social Determinants of Health Research Center, Research Institute for Prevention of Non-Communicable Diseases, Qazvin University of Medical Sciences, Bahonar blv., 34197-59811 Qazvin, Iran

**Keywords:** Anxiety, Primary dysmenorrhea, Menstruation pain, Eye movement desensitization and reprocessing, EMDR

## Abstract

**Background:**

Unpleasant experiences of dysmenorrhea can lead to increased anxiety. The anxiety associated with dysmenorrhea is a pain-related anxiety which might reduce the efficacy of medication as well as enhance the perception of pain. The present study evaluated the efficacy of eye movement desensitization and reprocessing (EMDR) in reducing anxiety among female university students with primary dysmenorrhea.

**Methods:**

In this randomized controlled trial, 88 female university students were recruited from April 2019 to February 2020. Eligible participants were selected by convenience sampling and were allocated into study groups (44 individuals in the intervention group and comparison group) using balanced block randomization. The final sample comprised 78 participants who completed the study (39 individuals in each group). Data were collected using the Spielberger State-Trait Anxiety Inventory, Subjective Units of Distress Scale, and Validity of Cognition Scale before the intervention and at the time of the first menstrual period after completion of the intervention. The intervention group received EMDR in two individual interventional sessions which lasted approximately one hour. Data analysis was performed using analysis of variance with control of covariance method at a significance level of 0.05.

**Results:**

The results of the study showed that EMDR did not have a statistically significant effect on State-Trait Anxiety of patients with dysmenorrhea (*p* > 0.05). Based on the Cohen’s *d* effect size of 0.06 for state-anxiety, -0.01 for trait-anxiety, and partial eta square less than 0.059 for both uncorrected and corrected models, the intervention was within a trivial effect.

**Conclusion:**

EMDR intervention did not have a statistically and clinically significant effect on State-Trait Anxiety of patients with dysmenorrhea. Therefore, the efficacy of EMDR in treating dysmenorrhea-related anxiety remains inconclusive.

*Trial registration* IRCT20180823040851N2 on 2019-02-09.

## Background

Dysmenorrhea is the most common type of recurrent pain that occurs as acute pelvic pain during menstruation [1]. According to the underlying pathological mechanism, there are two types. Primary dysmenorrhea is the presence of painful menstruation in the absence of provable pelvic disease. Secondary dysmenorrhea is when pathological pelvic problems occur such as endometriosis and pelvic inflammatory disease, or pelvic leiomyoma [2]. The initial onset of primary dysmenorrhea usually begins one to two years after menarche and with the onset of ovulatory cycles [2]. Primary dysmenorrhea is a painful uterine cramp that is felt just before or during menstruation in the lower abdomen [1]. Regardless of culture and geography, the results of various review studies have reported a prevalence of primary dysmenorrhea in 16–91% of women of reproductive age [3–5], with moderate to severe pain in 40% [6]. Dysmenorrhea begins to decline after the age of 30 years and significantly after the age of 35 years [2]. Therefore, women experience this pain for a significant length of time in their lives.

Individuals react differently to pain regardless of the cause of the pain [7]. One of these reactions has been referred to as ‘pain-related anxiety’ which is considered as a new area of pain-related research [8]. Although the perception of pain and emotional anxiety are processed in higher brain structures, including areas of the cerebral cortex, early research on pain and anxiety was largely carried out separately [9]. However, in practice, it has been shown that pain and anxiety may interact with each other, and a positive association has been observed between them [10]. The results of Means-Christensen et al.’s study (2008) also reported that patients seeking primary care treatment with symptoms of pain had lower mental health status, and higher levels of depression, social anxiety, and post-traumatic stress disorder. Similarly, patients with anxiety or depression report greater pain [11]. Dysmenorrhea is a chronic cyclic pain, which has a positive and significant association with psychological disorders such as depression, anxiety, and stress as reported in a recent systematic review [12]. Therefore, dysmenorrhea might cause pain-related anxiety but is a personal reaction.

Mood disorders, depression or anxiety disorders can have different effects on the transmission and perception of pain. The association between mood disorders and pain is a bidirectional association, with each appearing to act as a risk factor for the other. Depression and anxiety are associated with increased perception of pain severity, whereas prolonged duration of acute pain leads to an increase in mental health disorders, including anxiety [13]. Pain-related anxiety is a predictor of pain-related behaviors during treatment [7]. In primary dysmenorrhea, depression and anxiety due to the impact of pain on social and occupational functions can reduce the response to medication and also enhance the perception of pain [14].

At present, a variety of pharmacological and non-pharmacological methods are used to treat dysmenorrhea. Painkillers such as acetaminophen, cyclooxygenase (COX)-inhibiting drugs such as celecoxib and contraceptive pills are the main pharmacological treatments for primary dysmenorrhea with a failure rate of 20–25% [15]. This failure of medication might be due to psychological factors, which can exacerbate the pain [16]. For this reason, despite various treatments available for the treatment of primary dysmenorrhea, some cases of primary dysmenorrhea cannot be controlled [17]. Given the potential role of psychological distress, such as unpleasant experience with the previous menstruation and positive and significant relationship between anxiety and primary dysmenorrhea, the effectiveness of psychotherapy-based and behavioral interventions requires further examination [15]. Behavioral interventions are based on the assumption that psychological and environmental factors affect physiological processes. The purpose of these interventions is initially to change individuals’ thoughts or cognition which consequently changes behavior [15, 18]. A systematic review by Proctor et al. (2007) reported positive results of behavioral interventions to relieve dysmenorrhea pain [15].

Given the role of trauma from previous unpleasant experience of dysmenorrhea [19] and the positive relationship between anxiety and dysmenorrhea [12], the impact of psychological methods to manage anxiety can be considered as a non-pharmacological method of anxiety management related to dysmenorrhea. Among the non-pharmacological treatments for anxiety, there is a form of psychotherapy called Eye Movement Desensitization and Reprocessing (EMDR), developed in 1987 by Francis Shapiro [20]. The main purpose of this technique is to identify negative thoughts and replace it with pleasant ones. EMDR therapy is a systematic, comprehensive, non-invasive, simple, and evidence-based treatment for unpleasant memories and related events. EMDR is a method used to treat anxiety, depression, chronic pain, phantom pain, somatization syndrome, and post-traumatic stress syndrome, and has shown promising results [19, 21–25]. The results of systematic review by Tesarz (2014) showed that the effectiveness of EMDR therapy in the treatment of patients with chronic pain is promising, and is safe with lasting therapeutic effects [26]. The success rate of this method in one to three sessions, depending on the severity of the trauma, is reported to be 84–90% [27]. The effectiveness of this method is greater than that of traditional therapies, where their success rate is less than 55% in 7–13 sessions [27]. Therefore, the main objective of the present study was to evaluate the efficacy of eye movement desensitization and reprocessing on anxiety among female university students with primary dysmenorrhea.

## Methods

### Design and participants

**Fig. 1:**
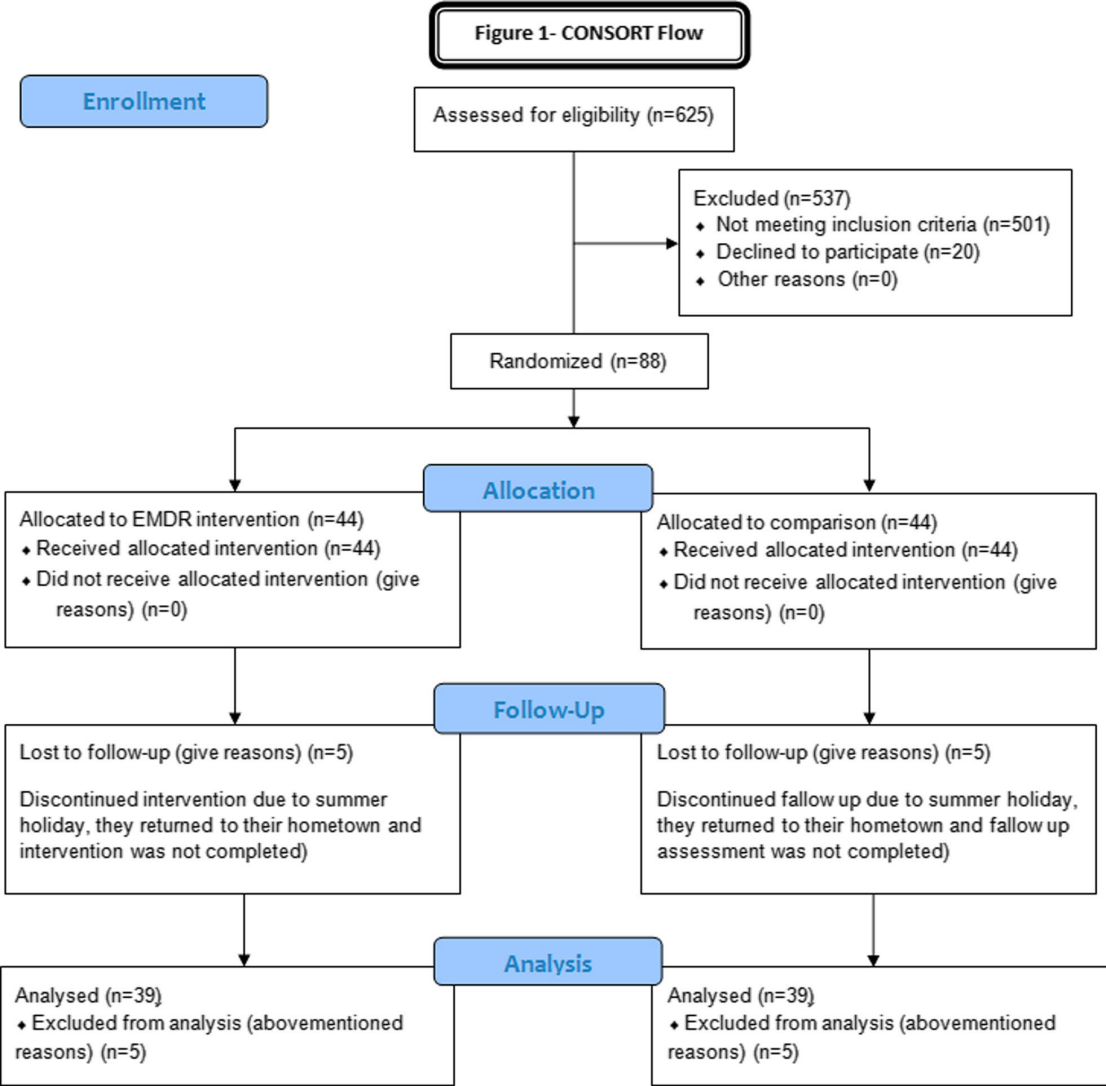
CONSORT flowchart

The present study was a randomized controlled trial carried out with female university students. It was prepared based on Consolidated Standards of Reporting Trials (CONSORT). The CONSORT Flowchart is presented in Fig. 1. The study participants comprised 88 female students. Single student girls aged 18 to 35 with moderate to severe primary dysmenorrhea (score above 4 on the Visual Analogue Scale) were eligible to participate in the study. The exclusion criteria included the presence of secondary dysmenorrhea (based on individuals’ previous reports of pelvic ultrasound examination), history of pelvic or abdominal surgery, history of physical and mental illness based on the individual’s statement, a history of seizures, strabismus, and vision problems, and the likelihood of graduation during the follow-up period. The girls’ dormitory and classrooms of the various faculties of university was research environment of the present study. The first author visited the dormitories during off-class hours and classes during breaks. In addition, an announcement for participation in the project was posted on the notice boards of dormitories and colleges.

### Estimation of sample size and sampling process

According to previous studies [28, 29] and considering the size of the effect of 0.25, study power of 80%, the first type of error of 0.05, sample size was estimated using G-Power software. The estimated sample size was 41 in each group. Considering the 10% probability of the loss of sample, 88 people were invited to participate. Eligible participants were selected by convenience sampling method and then randomly assigned to intervention or comparison groups.

### Random allocation procedure

The random allocation of participants into two groups utilized the balanced block randomization method. Balanced block randomization results in an equal number of participants assigned to each study group [30]. In the present study, block randomization was run within blocks of four. For example, given a block size of four, there are six possible ways to equally assign participants to a block (A: EMDR group; B: comparison group). All possible modes of the four blocks were written and numbered as follows: 1. AABB; 2. ABAB; 3. BBAA; 4. BABA; 5. ABBA; 6. BAAB. Consequently, in each block, two participants were assigned to the intervention group and two were assigned to the comparison group. The allocation sequence was prepared using the randomizer.org website before starting the study, with letter A for the EMDR group and the letter B for the comparison group. In order to conceal the allocation sequence, 88 opaque envelopes were prepared. The randomization sequence was written in separate sheets and placed in envelopes 1–88, respectively. Afterwards, the list of allocation sequences was destroyed.

### Outcomes and measures

The main outcome measure of the present study was the level anxiety among female university students with primary dysmenorrhea. Anxiety was assessed using the Spielberger State-Trait Anxiety Inventory (STAI). The STAI is used to assess state-anxiety and trait-anxiety, each with 20 items and rated on four-point Likert scales. Higher score indicates greater anxiety. State-anxiety showed good convergent validity with Taylor Manifest Anxiety Scale (TMAS) with a high correlation of 0.79 to 0.83 and the correlation between trait anxiety and list of affective traits has been reported from 0.52 to 0.58. Spielberger and Gonzalez have reported Cronbach’s alpha coefficient of State Anxiety Inventory and Trait Anxiety Inventory to be 0.92 and 0.90, respectively. In addition, retest State Anxiety Inventory were coefficients from 0.16 to 0.86, and for total, Cronbach’s alpha coefficient was 0.94 [31]. The Persian version of STAI has a Cronbach's alpha coefficient of 0.9 [32]. The STAI was completed at two time points (i.e., before the intervention and at the next menstrual period after intervention had finished).

In addition to STAI, Subjective Units of Anxiety or Distress Scale (SUD) and Validity of Cognitions Scale (VOC) were used to verify the EMDR process. The SUD is self-reporting scale with score ranges between 0 (no anxiety) and 10 (maximum anxiety). This scale has been used extensively in almost all behavior therapy techniques and clinical practices. Based on SUD, the individual assesses and reports the degree of discomfort or anxiety at each stage according to the request of the therapist or researcher [33]. The VOC or test of semantic difference in cognitions indicates an individual’s belief in a positive or negative cognition. The VOC is an eight-point self-report scale and assesses an individual’s self-recognition. At each stage and at the request of the therapist, the participant evaluates, grades, and expresses their level of belief about a sentence expressed by the individual and indicates the type of cognition they have in relation to the subject matter. If a positive cognition is desired, a score of zero corresponds to a lack of belief and a score of 7 corresponds to complete belief [33]. The SUD and VOC were procedural outcome variables which guided the researcher in conducting EMDR in each session. These scales were administered at the start and the end of each session. Also, for all participants a demographic and menstrual characteristics questionnaire including questions concerning age, age of menarche, education, field of study, history of taking painkillers was completed.

### Intervention

EMDR comprises eight essential phases. Several phases may be performed in one treatment session. The first phase includes taking the patient’s history, designing the treatment, preparing the patient, and evaluating the patient. The assessment phase includes setting the goal and setting the baseline responses, which are assessed by the patient’s comments on the SUD and VOC. Before starting treatment with EMDR for the first time, the therapist advises the patient to identify a safe place, image, or memory in which they feel comfortable and at ease, so that when they experience unpleasant feelings, the individual can imagine it and can endure those unpleasant feelings. In the third phase, desensitization is carried out to target the annoying emotions of the patient. The setting-up phase in the fourth phase focuses on cognitive reconstruction and reprocessing (installation phase). In the next phase, the remaining physical stress is evaluated. This phase is called physical scanning. The completion or closure phase includes a reciprocal report and is primarily designed to maintain the patient’s balance between sessions and is re-evaluated at the end. The therapist provides the patients with appropriate information and gives them adequate support in the seventh phase. The final phase is reassessment aimed to ensure the processing of all relevant old events [20, 33]. The intervention procedure is described below:

- Phase 1: To identify treatment target, the participant was asked to talk about their experiences of menstruation, dysmenorrhea and menstrual-related complaints, as well as the epicenter of the pain. The participant was also asked to specify a traumatic event regarding dysmenorrhea which disturbed them the most.
- Phase 2: For preparation, therapeutic targets of intervention were established. The procedure of EMDR therapy, its effects, and safety were explained. The participant’s concerns and questions regarding EMDR were addressed.
- Phase 3: In the assessment phase, the participant’s negative cognitions and their worst unpleasant memory about dysmenorrhea were identified. Participants were then asked to choose a positive cognition to be used to replace the negative cognition during the installation phase. They were encouraged to imagine having a less painful menstruation as a positive cognition.
- Phase 4: Desensitization targets the participant’s unpleasant feeling regarding dysmenorrhea. First their pre-treatment SUD and VOC scores were assessed. Eye movement sets were then used and the distressing memory of dysmenorrhea was processed. At the end of the phase, the participant was asked about their level of negative affect using the SUD scale.
- Phase 5: Installation focuses on strengthening the positive cognition to replace the negative cognition. The researcher tried to install the positive cognition of menstruation without dysmenorrhea in the participant’s mind. The participant was asked to provide their VOC score.
- Phase 6: Body scans assess and evaluate the physical tensions regarding dysmenorrhea. After installing the positive cognition, the participant was asked to hold in mind both the target event (dysmenorrhea) and the positive cognition (menstruation without dysmenorrhea) and then scan their own body mentally from top to bottom. Any remaining discomfort in the form of body sensation was then identified.
- Phase 7: In the closure phase, the participant attempted to reach a state of emotional equilibrium. They were informed about the possibility of having disturbing images, thoughts or emotions between the sessions.
- Phase 8: In the re-evaluation phase, the researcher ensured that all relevant events had been processed [34]. All phases can be conducted in each session. In the present study, the essential phases were conducted completely in first session [20, 33]. In the second session, Phases 4 to 8 were repeated to ensure the established effect of EMDR (which was acquired in the previous session based on VOC and SUD scores). The intervention was performed by the first author under the supervision of the third author who is qualified in EMDR. The first author learned how to perform the intervention, and then performed the first five interventions under careful supervision and in the supervisor’s presence. After obtaining the necessary competency, the intervention was performed independently. The intervention took place in rooms with suitable conditions (in the dormitory, counseling room and an empty room in the School of Midwifery Nursing) which was provided to the researcher to perform the intervention.

### Number of intervention sessions

To determine the appropriate number of sessions to perform the intervention, studies that used the EMDR method for chronic pain and anxiety were reviewed. The results of both systematic review of the effectiveness of EMDR in treating chronic pain [25] and anxiety disorder [35] showed that an average of six sessions were used. So, the maximum number of treatment sessions was selected to be six sessions to be held twice a week. The duration of each session was approximately one hour depending on the willingness and comfort of the participants [33]. Considering the fact that some individuals may respond to treatment before reaching the predefined number of sessions, it was decided that if they responded to the treatment (based on SUD and VOC results), the treatment would continue for another session and report the number of treatment sessions.

Participants in control group could use their usual dysmenorrheal pain-controlling methods such as using painkillers or non-pharmacological methods. They were asked to do the same as they normally did during the study period and to report the details to the research team. Those in the control group were also offered the option to try EMDR after completion of the study.

### Ethical considerations

The proposal of this research was approved by the institutional review board and ethics committee (ethics number IR.QUMS.1397.176). The study protocol was registered prospectively in the Iranian Registry of Clinical Trials with referee number of IRCT20180823040851N2 on 2019-02-09. It is noteworthy that the present study was conducted in line with another project to investigate the effect of EMDR on the severity of dysmenorrhea pain. The main concern of the present study was anxiety related to dysmenorrhea.

After obtaining the necessary permits, the individuals were invited to participate in the study. Necessary explanations were given to the research participants about the purpose of the research, the type of work and the assurance about the confidentiality of the information. Written consent was obtained from the participants. Participants were told that they could withdraw from the study and use common treatments and remedies whenever they did not want to continue or if they had severe pain or discomfort.

### Statistical analysis

Data analysis was performed using Stata version 14.0 (Stata Corp LLC, Texas, USA). Categorical data were described using absolute and relative frequencies and quantitative data were described using means and standard deviations (SDs). Between-group comparison of baseline characteristics utilized the proposed method of Imbens and Rubin by considering the standardized means difference less than 0.25 for continuous quantitative variables and the risk difference index less than 10% for qualitative variables [36]. The distribution of quantitative data (anxiety, SUD and VOC scores) was assessed using histograms, comparing central and dispersion indices, and the Shapiro-Wilks test. Due to the normal distribution of anxiety scores within study groups, one-way analysis of variance (ANOVA) was used to compare anxiety scores after intervention, in an uncorrected model. In addition, covariance analysis (ANOVA-ANCOVA) was performed to compare groups based on the corrected anxiety score to control the effect of the average pre-test anxiety score. Due to the non-normal distribution of SUD and VOC scores, the Wilcoxon test was used.

The size of the effect, including the partial eta square, the mean difference (MD), and the standardized mean difference (SMD) based on Cohen’s *d* were used. Cohen’s *d* 0.2–0.5 is considered “small” effect size, 0.5–0.8 represents a “medium” effect size, and greater than 0.8 is a “large” effect size. Moreover, partial eta-square is interpreted as: 0.010–0.059: small, 0.060–0.139: medium and more than 0.140: large [37]. MD was interpreted in terms of minimal clinically important difference (MCID) for STAI. Knudsen et al. (2019) reported the least significant clinical change of 10 on the Spielberger State-Anxiety Inventory [38]. Therefore, the MCID for the anxiety score in the subscales of STAI was considered to be at least 10. Effect sizes were reported with 95% confidence interval. Significant levels of less than 0.05 were considered.

## Results

In the present randomized controlled trial, 88 female students with primary dysmenorrhea participated and were assigned randomly into two groups of 44 as an intervention and comparison group. During the study, ten individuals were lost from the study (five in each group). Withdrawal was due to difficulty in attending the meetings or failing to complete subsequent questionnaires because the participants were busy taking final exams and do not have time to continue cooperation. Therefore, the final sample comprised 78 people (39 in each group) who participated until the end of the study. Table 1 shows the distribution of demographic variables and menstrual characteristics of the two groups. According to Table 1, age and severity of menstrual pain were slightly different between the two groups. Therefore, the effect of these variables besides the pre-intervention anxiety score was investigated as covariance in the corrected model.

**Table 1:** Distribution of qualitative and quantitative variables of demographic characteristics of participants by two groups of intervention and comparison

Variable	Intervention group (39 people)		Comparison group (39 people)	
	N	%	N	%
Economic situation of the family				
Good	15	30.8	12	38.5
Medium	24	61.5	27	69.2
Menstrual pain relief methods				
Not used	0	0	1	2.6
Pharmacological method	14	35.7	17	43.6
Non-pharmacological method	5	12.8	3	7.7
Both	20	51.5	18	46.2
Use of relief method in the last 2 months				
Yes	32	82.1	34	87.2
No	7	17.9	5	12.8

Quantitative variables	Mean	SD	Mean	SD
Age (year)	21.49	1.72	22.26	2.99
Age of menarche (year)	12.9	1.73	12.85	1.53
Educational semester	4.44	2.9	4.97	3.22
The severity of menstrual pain (VAS 0–10)	6.52	1.3	6.05	1.34
Menstrual pain duration (days)	2.23	0.9	2.36	0.98

### Effectiveness of intervention

The effect of EMDR intervention was investigated in the uncorrected model using one-way ANOVA and in the corrected model using ANOVA-ANCOVA. The results are presented in Table 2. Corrections were made for pre-test anxiety scores, menstrual pain severity, and age for both State and Trait subscales. The results of the study showed that the intervention did not have a statistically significant effect on State-Trait anxiety of the patients with dysmenorrhea (*p* < 0.05). Cohen’s *d* of 0.06 for state-anxiety and − 0.01 for trait-anxiety and the partial eta square of less than 0.059 implied that the effect of intervention was negligible. In none of the corrected and uncorrected models, the minimal clinically important change was not achieved.

**Table 2:** Results of analysis of variance–covariance (ANOVA-ANCOVA) to investigate the effect of EMDR on dysmenorrhea-related anxiety based on State and Trait subscales

Outcome	Model*	Time point	Intervention (n = 39)		Comparison (n = 39)		Mean difference		Cohen’s *d*		Partial eta square	*p* value
			Mean	SD	Mean	SD	Point	95% CI	Point	95% CI		
State anxiety	Crude	Pre- intervention	46	12.34	43.56	13.24	2.44	− 3.34; 8.21	− 0.19	− 0.64; − 0.25		
		Post-intervention	45.49	10.34	44.87	11.78	0.62	− 4.39; 5.62	0.06	− 0.39; 0.5	0.001	0.81
	Adjusted	Post-intervention*	44.84	8.88	45.51	8.8	− 0.67	− 4.68; 3.33	− 0.08	− 0.52; 0.37	0.001	< 0 .001
		Post-intervention**	44.66	8.93	45.69	8.93	− 1.03	− 5.09; 3.03	− 0.12	− 0.56; 0.33	0.003	0.29
		Post-intervention***	44.64	8.97	45.71	8.97	− 1.07	− 5.18; 3.04	− 0.12	− 0.33; − 0.56	0.004	0.72
Trait anxiety	Crude	Pre-intervention	44.61	11.07	43.87	11.78	0.74	− 4.41; 5.9	− 0.07	− 0.51; − 0.38		
		Post-intervention	43.9	10.68	44	10.68	− 0.10	− 4.92; 4.72	− 0.01	− 0.45; 0.43	< 0.001	0.97
	Adjusted	Post-intervention*	43.65	7.71	44.24	7.71	− 0.59	− 4.07; 2.89	− 0.08	− 0.52; 0.36	.002	< 0.001
		Post-intervention**	43.50	7.78	44.39	7.78	− 0.88	− 4.42; 2.65	− 0.11	− 0.56; 0.33	.003	0.35
		Post-intervention***	43.56	7.87	44.34	7.87	− 0.77	− 4.37; 2.83	− 0.10	− 0.54; 0.35	0.003	0.70

The crude model was analyzed using one-way ANOVA, adjusted models were analyzed using ANOVA-ANCOVA

*Adjusted for baseline anxiety score

**Adjusted for baseline anxiety score, pain intensity

***Adjusted for baseline anxiety score, pain intensity and age

### Required number of treatment sessions

SUD and VOC are procedural outcome variables which guide the researcher in conducting EMDR in each session. These scales were used at the start and end of each session. Approximately 90% of the participants reached acceptable levels of SUD and VOC in the first session and their score remain unchanged in the second session which confirmed the adequacy of interventional sessions. The SUD and VOC data did not have a normal distribution, so the Wilcoxon nonparametric test was used to compare the scores before and after the intervention. The results of the Wilcoxon test showed a significant difference between the SUD and VOC scores before and after intervention. This verified the adequacy of the number of intervention sessions (Table 3).

**Table 3:** Median distribution and interquartile range between the scores of subjective anxiety and cognitive validity scales, before and after the intervention in the two intervention groups

Variable	Before intervention	After intervention	The significance level of the Wilcoxon test
	Median (IQR)	Median (IQR)	
Subjective anxiety (0–10)	6 (8–5)	0 (0)	< 0.001
The validity of cognition (0–7)	6 (6–5)	0 (0)	< 0.001

IQR: interquartile range

## Discussion

The present study is the first to examine the effect of EMDR in reducing anxiety related to dysmenorrhea using a randomized clinical trial design with a concurrent comparison group. The results of the study showed that the intervention did not have a statistically and clinically significant effect on state and trait anxiety of the participants with primary dysmenorrhea. Although the measure of effects, including MD, SMD and the partial eta squared showed a slight increase after the correction of the results, the effectiveness of EMDR in reducing dysmenorrhea-related anxiety remains inconclusive. In addition, narrow confidence intervals for standardized mean differences in all models was within the scope of the inconsiderable effect, which could be a sign of the conclusive results and the adequacy of the sample size in evaluating the effect of this intervention. Due to the fact that the minimal clinically significant change was determined at least 10 points changes [38], the intervention was not clinically effective.

EMDR is a method used to treat anxiety, depression, chronic pain, imaginary pain, somatization syndrome, and post-traumatic stress syndrome, and has shown promising results [19, 21–25]. It has also shown effective results in significantly reducing anxiety in patients with heart attack [39] and anxiety of adolescents suffering from thalassemia [21], and patients’ anxiety awaiting cardiac catheterization [40]. In addition, a systematic review study in summarizing the results of clinical trial studies reported positive effects of EMDR intervention in the treatment of emotional trauma and other adverse life experiences [41]. Also a recent meta-analysis of randomized controlled trials showed that EMDR is efficacious for reducing symptoms of anxiety, panic, phobia, and behavioral /somatic symptoms [42]. Despite previous promising results, EMDR therapy did not reduce anxiety associated with dysmenorrhea. While the treatment was adequate based on SUD and VOC scores, no significant effect was observed on state and trait anxiety associated with dysmenorrhea. One possible reason could be because of differences in the tools used. Some previous studies have used Beck Anxiety Inventory (BAI) [21, 39, 40], while the present study used the STAI. Given that obtaining a score of 32 to 53 on the State-Trait Anxiety Inventory means moderate anxiety. [31], all participants in the present study had moderate anxiety. However, participants in previous studies had high level of anxiety. Therefore, another difference could be the level of anxiety between participants. Previous studies have shown that EMDR has been an effective way to treat severe anxiety, but given the present findings, it may not be effective enough for moderate anxiety. Also, this might also be due to lack of specific scale to assess dysmenorrhea-related anxiety. The STAI assesses general anxiety. As dysmenorrhea and menstruation might exert specific situations for a female to be anxious from, these aspects might not be assessed properly using a general anxiety scale. Despite the fact that the frequency of intervention sessions was selected based on the results of SUD and VOC scores of participants, two sessions might be insufficient to change the STAI scores. Results of a current systematic review confirmed the lack of high quality studies regarding the effectiveness of EMDR on different mental health outcomes including unpleasant traumas [42] and other mental health problems [43]. As the effectiveness of EMDR in treating dysmenorrhea-related anxiety remained inconclusive in the present study, further studies with high methodological quality and greater sample sizes are needed.

### Limitations

Having a randomized controlled trial design and adequate sample size were the strengths of the present study, but the study also has limitations that should be considered in interpreting the present findings and designing future studies. Limitations of the present study included having subjective outcomes assessed by patients’ self-report, lack of specific scale to assess anxiety related to dysmenorrhea, and lack of placebo group to control the psychological effect of intervention (and so, lack of blinding). Other limitation of current study is that the practitioner who conduct the EMDR was not an accredited and experienced EMDR therapist. While the practitioner learned how to perform the intervention from an experienced EMDR therapist, and then performed the first EMDR sessions under careful supervision EMDR therapist, but this point should be considered as a limitation for current study.

### Clinical implication

To best of our knowledge, present study is the first which investigate the effect of EMDR in reducing anxiety among female university students with primary dysmenorrhea. While effectiveness of EMDR technique is established in treatment of anxiety, depression, chronic pain, phantom pain, somatization syndrome, and post-traumatic stress syndrome, and has shown promising results in previous literature [19, 21–25], we did not find a statistically or clinically significant effect on primary-related anxiety. As participants in the current study experienced moderate levels of anxiety as well as limitations considered, it seems that EMDR can be considered as complementary therapeutic technique for individuals with dysmenorrhea pain associated with high level of anxiety. Also, there did not appear to be any negative effects of using EMDR and it may be a better intervention than doing nothing at all given that the comparison group could still engage in their own psychological and/or pharmacological intervention in the present study.

## Conclusion

The EMDR intervention did not have a statistically or clinically significant effect on primary-related anxiety so cannot be recommended for further use in trying to lower anxiety level among female university students with dysmenorrhea at present. Further studies with high methodological quality and greater (as well as more representative) sample sizes and more intervention sessions are recommended as that might result in more conclusive results regarding the effectiveness of EMDR and whether it can reduce anxiety among individuals with primary dysmenorrhea.

## Data Availability

Materials and dataset used and analyzed during current study are available from the corresponding author on reasonable request.

## Abbreviation

EMDR: Eye movement desensitization and reprocessing
